# Acknowledgment to the Reviewers of *Antibiotics* in 2022

**DOI:** 10.3390/antibiotics12020185

**Published:** 2023-01-17

**Authors:** Antibiotics Editorial Office

**Affiliations:** MDPI AG, St. Alban-Anlage 66, 4052 Basel, Switzerland

High-quality academic publishing is built on rigorous peer review. *Antibiotics* was able to uphold its high standards for published papers due to the outstanding efforts of our reviewers. Thanks to the efforts of our reviewers in 2022, the median time to first decision was 13 days and the median time to publication was 33 days. Regardless of whether the articles they examined were ultimately published, the editors would like to express their appreciation and thank the following reviewers for the time and dedication that they have shown *Antibiotics*:
A. A. KorzhenkovKazuhiro P. IzawaA. N. M. Mamun-Or-RashiKazuto TajiriA. N. M. Mamun-Or-RashidKegan Romelle JonesAabir BanerjiKeila Y. Acevedo-VillanuevaAalfin EmmanuelKenchappa PrashanthAbad KhanKenji HayashidaAbdelrahim HassanKennedy ChahAbdorreza Mohammadi NafchiKenneth GenoveseAbdul BasitKenza E. BenzeroualAbdul Haseeb HaseebKerstin StinglAbdul Rehman PhullKevimy AgossaAbdulaziz AlhazmiKevin MeestersAbdulkarim HasanKhalid S. IbrahimAbdullah KhanKhan KhalidAbdur RahmanKieu The Loan TrinhAbele KuipersKi-Kwang OhAbhijit SarkarKim StanfordAbhishek ChauhanKimberly ShepardAbhishek DeyKira V. VyatkinaAbhishesh MehataKirill V. ZaitsevAbia Akebe Luther KingKirk P. ManfrediAbid UllahKishneth PalanivelooAbolfazl Dashtbani-RoozbehaniKit Leong CheongAbraham Fikru MechessoKitherian SahayarajAbu Sayed ChowdhuryKlaus BrandenburgAbul Kalam AzadKlaus HantkeAbuzer AliKlejda HarasaniAdam KawalekKodjovi D. MlagaAdam KowalczykKomwit SurachatAdam MatkowskiKonstantina BitchavaAdam PyzikKonstantina DafopoulouAdam ValcekKonstantina FotouAdeel MalikKonstantinos KatsasAdina FésüsKonstantinos LiarasAdina FrumKonstantinos PontikisAditya Reddy KolliKonstantinos TsikopoulosAditya WaghKoteswara Yerra RaoAdnan A. IsmaielKousalya PrabaharAdnan Muhammad ShahKovy Arteaga-LiviasAdrián González-CastilloKrishna C. ChintaAdrian John BrinkKrishna KurthkotiAdrian ManKrishna SharmaAdrian Maximilian MacriKrishna YadavAdriana CostaKrishnamoorthy SrikanthAdriana Cristina UrcanKristin SurmannAdriana De Jesus Inácio BelasKristjan BregendahlAdriana HanganKrisztina M. Papp-WallaceAdriana IsvoranKrithika RajagopalanAdriána LiptákováKrupa NavalkarAdriana MorarKrutika MediwalaAdriana TrottaKrzesimir CiuraAdriano C. CoelhoKuldeep GuptaAdrija HajraKun YangAfzal ShaikKunal AjmeraAgata PiecuchKunal PalAgnes NagyKundan MishraAgnieszka GrabowieckaKurt NaberAgnieszka HerosimczykKusum DhakarAgnieszka KwiatekKyaw Thu AungAgnieszka MroczekLacramioara OchiuzAgnieszka NecelLaila ZikoAgnieszka Olejnik-SchmidtLakshmanan GovindanAgnieszka RazimLalit GolaniAgnieszka Ulatowska-JarżaLamine Baba-MoussaAgus LiandiLan ZhangAhmad G. HegaziLara SaviniAhmad Rois MansurLarisa IlinaAhmed Abu-AwwadLarry H. DanzigerAhmed Atef MesalamLars MalmströmAhmed Eisa ElhagLászló FodorAhmed ElbestawyLaszlo RadoczAhmed FaramawyLatefa BiskriAhmed HamedLaura CerqueiraAhmed KarmaouiLaura De PlanoAhmed M. DebelaLaura FolgoriAhmed M. ElissawyLaura Miller ConradAhmed M. SayedLaura Naemi WaltiAhmed NabawyLaura Soldevila-BoixaderAhmed NafisLaura VeschettiAhmed Neamat-AllahLaure Peyro-Saint-PaulAhmed Ud DinLauren N. ParsonsAida DuarteLaurent BiglerAida MetoLaurent ChiarelliAida PetcaLawrence MugishaAikaterini-Elisavet DoufexiLaychiluh MekonnenAirat KayumovLe Phuong NguyenAitor Fernandez-NovoLee Lee TengAitor Rizo-LiendoLee NguyenAitziber Antoran DiazLeena AwawdehAjay Kumar GautamLei HeAjay Suresh AkhadeLei ZhaAjda MoškričLeila HojatAjit SinghLeire VirtoAkebe Luther King AbiaLenka MicenkováAkhtar AliLeonard KoolmanÁkos JerzseleLeonardo BrunettiÁkos PethőLeonardo PaganiAkram AshamesLeonor MeiselAkshaya BhagavathulaLetizia AngiolellaAlakananda DasLi XiaoAlbert BolatchievLiang ChenAlbert FiguerasLiang ZhuAlberto AntonelliLiangjun XiaAlberto BerardiLianping YangAlberto De BiaseLiča LozicaAlberto Enrico MaraoloLichun HeAlberto EvangelistaLicia PantanoAlberto GregoriLidia GavicAlberto Jorge Oliveira LopesLidia Hanna MarkiewiczAlberto MellaLidia Ruiz-RoldánAlberto PisperoLidija BarisicAldo NicosiaLigen YuAldo Proietti-JuniorLiisa KaartinenAleia F. CamposLiisa VeerusAlejandro DashtiLilia Coronato CourrolAlejandro Garrido-MaestuLin QiuAlejandro Miguel Figueroa-LópezLinda BesterAlejandro Pérez-LariosLinda HouhamdiAlejandro PiscoyaLing LiuAlejandro PlascenciaLinna AnAlejandro Speck-PlancheLinus OlsonAleksandar PavicLisa BenjaminAleksandra J. BorekLisanework E. AyalewAleksandra Kawczyk-KrupkaLiudmila LeppikAleksandra KocotLiviu Mihai IrimiaAleksandra M. BondžićLiviu SteierAleksandra MarciniakLoganathan PonnusamyAleksandra Maria KocotLoh Teng-Hern TanAleksandra P. PetrovićLong Chiau MingAleksandra Petrovic FabijanLoredana RadoïAleksandra TroscianczykLoredana Sabina Cornelia ManolescuAleksandra UzelacLorena Elena MeliţAleš ObrezaLorenzo AntoniniAlessandra AmmazzalorsoLorenzo FraileAlessandra PiccirilloLorenzo OnoratoAlessandra SaporitiLori BanksAlessandro FeolaLorina Badger-EmekaAlessandro RussoLoris PintoAlessia CatalanoLoukia EkateriniadouAlessio BuonavogliaLu LiAlexanda MzulaLu LuAlexander A. PugovkinLu YangchengAlexander A. Yu. Vasil’kovLuan Luong ChuAlexander BunevLubna Abu-NiaajAlexander HeißLuca AnsaloniAlexander HynesLuca FreddiAlexander KrivoruchkoLuca PalazzoloAlexander MankinLuca Paolo WeltertAlexander Nesterov-MuellerLucia BírošováAlexander ShcherbakovLucía Rodríguez-LópezAlexandra Cristina MunteanuLuciano NakazatoAlexandra KhlyustovaLucia-Roxana CojocAlexandra PeregrinaŁucja Zielińska-TomczakAlexandra TabaranLucjan WitkowskiAlexandre Gonzalez-RodriguezLudger StändkerAlexandre VetcherLudwig SedlacekAlexandru VlaicuLuigi PrincipeAlexey S. TreninLuigi RuggieroAlexey V. RakovLuis Alberto Ortega-RamirezAlexey VasilchenkoLuis Antônio EsmerinoAlexios Vlamis-GardikasLuís C. N. Da SilvaAlfonso Del Arco JiménezLuis Cláudio Nascimento Da SilvaAlfredo Téllez-ValenciaLuis Daniel Goyzueta MamaniAli Adem BaharLuis G. Sequeda-CastañedaAli AhmedLuisa SorlíAli IrfanLuka JurinovićAli OzcanLukas SchwarzAlice ChateauŁukasz NawackiAlice Elena GheneaŁukasz SobottaAlicia Gomez-LopezŁukasz TomczykAlicja WęgrzynLuminita Gabriela MarutescuAlida VelooLuminiţa Maria NicaAline Chiodi BorgesLyn Li LimAline Maria Da SilvaMaarten De MolAlireza ShoariMadan VermaAlirica SuarezMadelon L. FinkelAlkiviadis VatopoulosMagda JanalikovaAlok MishraMagdalena BartnikAlona Keren-PazMagdalena DunowskaAlperen DegirmenciMagdalena FlorekAlvin Qijia ChuaMagdalena Kizerwetter-SwidaAmaç Fatih TuyunMagdalena RauschAmal A. M. ElgharbawyMahbubul MatinAmal SabaMahipal KesawatAman KumarMahmoud Abo-AshourAman Ullah KhanMahmoud AzzamAmanda ChamiehMahmoud BendaryAmanda PacholakMahmoud MadkourAmanda RoomeMahmoud Mostafa AzzamAmandeep SinghMainak MustafiAmani DiremMainul HaqueAmaniel KefleyesusMaira GoytiaAmarinder Singh SinghMaisem LaabeiAmbikapathi NivethaMaitreyee MukherjeeAmbuga BadariMaja C. PagnaccoAmeer Fawad ZahoorMaja HitlAmelia Ramon-LopezMaja VelhnerAmer Hayat KhanMajid KomijaniAmeya KulkarniMalcolm PageAmin Talebi Bazmin AbadiMałgorzata CzatzkowskaAmir AshoorzadehMalgorzata KolpaAmira MoawadMałgorzata Kosińska-PezdaAmit JaiswalMalgorzata KoziolAmit KumarMałgorzata OlejnikAmit Kumar Singh GautamMałgorzata RudnickaAmit PantMałgorzata WrzosekAmit RaiMalik MaazaAmit SharmaMallique QaderAmit TripathiMamoon AldeyabAmiya Kumar SahooMana RaoAmjad Islam AqibManabu KuroboshiAmmar AlmaaytahManish KumarAmmar BaderManjusha LekshmiAmna ParveenManlio BarbarisiAmr Ahmed El‑ArabeyManmeet SinghAmrinder SinghManohar KugajiAmrita SarkarManohar RadhakrishnanAmy CroweManoli ZubiturAna AmaroManomi PereraAna AzevedoManon LaunayAna Lúcia F. PortoManuel Casal-GuisandeAna M. SahagunManuel CórdobaAna M. Sahagun PrietoManuel E. Machado-DuqueAna Maria UdreaManuel MadrazoAna MoragasManuel San AndresAna PerisinManuela CeccarelliAna Rita Xavier De Jesus GameiroMao HagiharaAna Šešelja PerišinMarc KoyackAna Sofia OliveiraMarc OlivaAna TomasMarc StadlerAna TomićMarc SturgillAna Vujačić NikezićMarcel F. KunrathAnahi DreserMarcel ManziAnand ManoharanMarcello CasertanoAnanda TiwariMarcelo Palma SirciliAnandrao Ashok PatilMarcia R. LeeAnant JainMarcia Regina FranzolinAnas ShamsiMarcia SaraivaAnastasia SpiliopoulouMarcin Bartłomiej ArciszewskiAnatoly N. VereshchaginMarcin Gra̧zAnatoly VereshchaginMárcio José De Abreu RodriguesAnca CarjeMarco ClericuzioAnca MareMarco FerrariAnca Niculina CadinoiuMarco ScocchiAnca StanaMarco TjioeAnca ZguraMarco Túlio Pardini GontijoAnca-Maria ArseniuMarcos Veiga Dos SantosAncuța ChetrariuMarcus Tullius ScottiAnderson Oliveira SouzaMarek MuriasAndré ChwalibogMargherita IzziAndré Conceição PereiraMari H. OgiharaAndrea BalliniMaria Angeles RojoAndrea BossoMaria Angelica GuimarãesAndrea BuscaroliMaría Auxiliadora Dea-AyuelaAndrea ButeraMaria BagattiniAndrea CassoniMaria BarsanAndrea Clemente PalmierMaria BartolomeuAndrea ConaMaria C. TeixeiraAndrea FulgioneMaría CeleiroAndrea GiacomettiMaria D’AccoltiAndrea Mariel SansoMaria De Lurdes CristianoAndrea MarinoMaria De OliveraAndrea MescolaMaria Elena Velázquez-MezaAndrea Micke MorenoMaria Eleni SakavitsiAndrea Moreno SwittMaria Evarista Arellano-GarciaAndrea RagusaMaría Guadalupe Frías-De-LeónAndrea RónaváriMaría GuembeAndrea SanchiniMaría Isabel Rodríguez LópezAndréanne LupienMaria Izabel A. CavassimAndreas Eugen EnzMaría Jesús Serrano AndrésAndreas NeffMaría José Cubero PabloAndreas Von Ameln-MayerhoferMaria KantereAndreea MogaMaria KourtiAndreea PricopieMaría LópezAndreia GarcêsMaria MatuzAndrés González SantanaMaria Melissa Gutierrez PachecoAndrew ClarkMaria Miklasińska-MajdanikAndrew CookeMaria NikolovaAndrew Jason CohenMaria Pia FerrazAndrew MeadMaria RajskaAndrew MurphyMaria SzymankiewiczAndrew OzgaMaría Teresa García-SanzAndrew T. LucasMaria Teresa MascellinoAndrey SgibnevMaria VitaleAndrzej CzyrskiMaría Vivero-LopezAndrzej KurylakMaria ZbrunAndrzej ZiębaMariaevelina AlfieriAneela Zameer DurraniMariam HassanAneliya MilanovaMariana BusilaAneta NowakiewiczMariana FerreiraAngel León-BuitimeaMariana María HuertaAngela BentonMariangela FedelAngela CesaroMarianna A. PatrauchanAngela Correa Freitas-AlmeidaMarianna MeschiariAngela Di SommaMarie HoarauAngela MontoneMariia NesterkinaAngela QuirinoMarija IvanovAngela RepanoviciMarija JozanovićAngela RizziMarija MarinAngela SalimMarijana SokolovicAngela TartagliaMarina CaldaraAngela WitteMarina JovanovićAngélica Villarruel-LópezMarina NisnevitchAngeliki KatsafadouMarina P. IsaevaAngelita Maria StabileMarina PapaianniAngelo BañaresMarina SofiaÂngelo LuísMarina SpinuAnida Maria BabtanMarina SpînuAniello AlfieriMarina StrakhovskayaAniello MaieseMarino VilovićAnil KumarMario D'IncauAnil PoudelMario Ferreira Conceição SantosAnil TharappelMario Quezada-AguiluzAnisha D’souzaMariola BochniarzAnita KotwaniMarios KrokidisAnja Kathrin JaehneMarios SpanakisAnka Trajkovska PetkoskaMarita MeurerAnke OsterlohMarius Belmondo TinchoAnkit PandeyaMarius ColinAnkita PunethaMarius StefanAnkur KaushalMarjan Wouthuyzen-BakkerAnna Baturo-CiesniewskaMarjeta TerčeljAnna BeniniMark ForsythAnna BielenicaMark G. MoloneyAnna BläckbergMark LawsAnna Duda-MadejMark T. McIntyreAnna ElkinaMarko BaškovićAnna JanowiczMarko GranićAnna KasprzykMarko TarleAnna Lenart-BorońMarko Z. MladenovićAnna ŁepeckaMarkus WeigelAnna M. F. MarinoMarkus ZeitlingerAnna MajewskaMarlene BenchimolAnna Maria SchitoMarta ArsuagaAnna OgrodowczykMarta CarvalhoAnna Paradowska-StolarzMarta DecAnna RozaliyaniMarta Helena BranquinhaAnna RóżańskaMarta Hernández-GarcíaAnna SiemiątkowskaMarta JaskulakAnna Szczerba-TurekMarta KsiazczykAnnalisa De SilvestriMarta LaranjoAnne HulinMarta Martínez-GuitiánAnne-Grete MärtsonMarta Sena VelezAnne-Laure RouxMartha E. WindelsAnne-Marie Perchec MerienMartín A. MaraschioAnnie Frelet-BarrandMartín Fernández-FerroAnnie Lee See MunMartin ŠímaAnoop Narayanan VadakkepushpakathMartin SoustAnroop NairMartina Baiardo RedaelliAnselm JordaMartina JuzbašićAnte PrkićMartina Srajer GajdosikAnthony Pokoo-AikinsMartyna MroczyńskaAnthony SlateMarwa ElgendyAnthony William ColemanMary B. FaroneAnton P. TyurinMary-Lorène GoddardAnton TyurinMaryna StasevychAntonella Francesca SimonettiMasaaki MinamiAntônia Eliene DuarteMasafumi ZaitsuAntonija TadinMasahiro OgawaAntonino GentileMasaomi KurokawaAntonino LauriaMasayoshi ShinjohAntonio BevilacquaMasayuki MaedaAntonio BusquetsMasayuki SatakeAntonio CascioMashkoor MohsinAntonio Del VecchioMashooq Ahmad BhatAntonio KlasanMassimiliano Matteo PellegriniAntónio MachadoMassimiliano VerouxAntonio PinnaMatei LiliaAntonio RussoMatilde SierraAntonio Sarria-SantameraMatteo CalcagnileAntonios E. KoutelidakisMatteo MoriAntony T. VincentMatthaios Papadimitriou-OlivgerisAntu DasMatthew DonaduAnudishi TyagiMatthew Gavino DonaduAnuj KumarMatthew GrantAnuja PandeMatthieu EveillardAnupam SharmaMatúš DohálAnusak KerdsinMaura Téllez-TéllezAnush AghajanyanMauricio ErbenAnusha AdityaMauro Cesar Palmeira VilarApichart AtipairinMax Carlos Ramírez SotoApt. DachriyanusMax RyadnovApurva Tadimari PrabhakarMaxime PichonAravinthkumar JayabalanMaya RubtsovaArbi GuetatMaytham HusseinAref ZayedMayukh GuhaArif LuqmanMayur K. LadumorArifin Budiman NugrahaMazen SalehArijit SenguptaMd Abdus SoburArisekar UlaganathanMd Bashir UddinAristides HatjimihailMd Iqbal HossainArleta DrozdMd Maidul IslamArlette Suzy SetiawanMd Saiful IslamArmando Silva-CunhaMd Shafiullah ParvejArmandodoriano BiancoMd Sofiqul IslamArne KienzleMd Tanvir RahmanArne SimonMd Zohorul IslamArnold S. BayerMedana ZamfirAroonsri PripremMeer M. ChisthiArpad Mihai RostasMeera MoydeenArpron LeesombunMegha SharmaArshad RizviMehrdad ForoughArtem BelousovMeng HeArtem RozhinMeng PuArthitaya Kawee-aiMengmeng ZhengArtur KhannanovMenino CottaArtur ŚwierczekMeram AbdelrahmanArturo Susarrey-ArceMerlin MoniArtyom Dmitrievich ObukhovMert SudağıdanArulmani ThiyagarajanMeysam SarsharArun KumarMiaomiao ShiArun S. KharatMicael GonçalvesArunaksharan NarayanankuttyMicaela ÁlvarezArvind NegiMicahel HumeAseer ManilalMichael AlkanAshaimaa MoussaMichael AntonysamyAshish KothariMichael GotsbacherAshleigh S. PaparellaMichael HässigAshok J. TamhankarMichael J. HearnAshok K. ShakyaMichael MahanAshraf M. OmarMichael O'ConnorAshutosh P. DubeyMichael TellierAshwini ChauhanMichael TunickAsif NawazMichael V. JoachimAsif SukriMichal ĎuračkaAsim Ali YaqoobMichal KajsíkAssèta KagambègaMichal LetekAssiya El KettaniMichal NiemczakAstrid Paulitsch-FuchsMichal PiotrowskiAsyraf RizalMichal ShteinbergAthanasia VarsakiMichel DionAtheesha SinghMichela CorròAthina GeronikakiMichela GambinoAtmika PaudelMichela PuglieseAtsumasa KomoriMichele BoffanoAtsushi TogawaMichele Cosimo SantoroAtukuri DorababuMichele FioreAtul M. ChanderMiguel A. CevallosAung Kyaw ThuMiguel A. Rojas Montes Rojas MontesAura GabrielMiguel Ángel MorenoAura RusuMiguel Ángel Navas-MartínAusilia GrassiMiguel Ángel Ramos LópezAvinash PremrajMihaela NiculaeAvishek MitraMihai JuncarAwatef BéjaouiMihai NitulescuAxel G. GriesbeckMihail Lucian BirsaAya KoizumiMihajlo ErdeljanAyesha RahmanMihajlo JakovljevicAyman GradaMiho JeongAyman Hassan Abd El-AzizMijat BožovićAyman MahmoudMikashmi KohliBabafela AwosileMike ScottBabak PakbinMikhail A. DziadzkoBabu JosephMikhail BerlovBachir Raho GhalemMikhail KryuchkovBadrinath KakdeMikio TanabeBaharak Babouee FluryMilad HadidiBaicus AndaMilan ČižmanBalajee RamachandranMilan KojicBalasubramanian MalaikozhundanMilan KolářBaldorj PagmadulamMilica AćimovićBalik DzhambazovMilica BajceticBaohua ChenMiljana JovandaricBarbara BrognaMiłosz LewandowskiBarbara De FilippisMin WangBarbara GierlikowskaMinakshi GandhiBarbara KotMinato YokoyamaBarbara MognettiMing YangBarros Rosana RochaMinhui GuanBarry R. PalmerMin-Hui LiBartłomiej GrygorcewiczMin-woo HaBartłomiej ZieniukMinyon AventBasama MnifMirela ImreBashar HannawiMiri KrupkinBasil MathewMiriam Pascual BenitoBassam ElgamoudiMirjam GrobbelBassma ElwakilMirjana JerkicBasudeb MondalMirna MihelčićBaysal OmurMiro JukićBeata Kasztelan-SzczerbinskaMiroslav BenićBeata Kowalska-KrochmalMiroslav FajfrBeata M. Gruber–BzuraMiroslava Htoutou SedlakovaBeata Mlynarczyk-BonikowskaMirosław GiurgBeatrice CasiniMiryam Criado GonzalezBeatriz LimaMitja KrizmanBela KocsisMitsuoki KawanoBela NagyMitsuru FukudaBelaghihalli N. GnaneshMiyase GündüzBelen Perez-SolansMohamad PadriBeniamin Oskar GrabarekMohamad YasminBenjamín Valladares-CarranzaMohamed AbdelwahabBenno Ter KuileMohamed Al-FatimiBernadette Emoke TelekyMohamed El YaagoubiBernd-Alois TenhagenMohamed El-RaiyBettina WollankeMohamed FarghaliBhagyaraj EllaMohamed LounisBiagio RaponeMohamed OrabiBiagio SantellaMohamed SalaheldeenBianca Maria GoffredoMohamed ZommitiBianca Maria TihăuanMohammad AatifBilal TantryMohammad Amjad KamalBiljana M. Todorović-MarkovicMohammad Azam AnsarBinaya SapkotaMohammad H. AlyamiBindu SubhadraMohammad HasanBing HanMohammad HojjatiBing LiMohammad ImranBini GeorgeMohammad Jamshed SiddiquiBinod RayamajheeMohammad Javad NajafzadehBipin AdhikariMohammad Javed AnsariBiswadeep DasMohammad KamalBiswanath JanaMohammad MofidfarBiyensa Gurmessa DubiwakMohammad MohsenzadehBlanca F. Iglesias-FigueroaMohammad Tahir SiddiquiBlanka Szekely-SzentmiklosiMohammad Tajul IslamBo PangMohammad Tarequl IslamBobrek KamilaMohammad Yaseen AbbasiBo-Cheng TangMohammad ZubairBogdan IliescuMohammad-Salar HosseiniBogdan IorgaMohammed F. HamzaBogumil BryckiMohammed KaraBojana BeovicMohammed NaielBojana DanilovicMohammed Oday EzzatBonaccorsi Di Patti Maria CarmelaMohammed ZawiahBongyoung KimMohan KaruppayilBono LučićMohandoss SonaimuthuBorja Herrero De La ParteMohanned Naif AlhussienBouchra SoulaimaniMohd AdnanBounlom DouangngeunMohd Amir AfjalBo-Young HongMohd Hafiz ArzmiBożena TyliszczakMohd SaeedBranka PilicMohmed Isaqali KarobariBratko FilipičMohsen SoltanifarBrenda A. Silva-EspinozaMohsin KhurshidBrian GodmanMona I. ShaabanBrijesh M. ShahMonia CocchiBrooke K. DeckerMónica CarreraBruno Bueno-SilvaMónica Echeverry-RendónBruno Francesco Rodrigues De OliveiraMonica GulatiBuket Er DemirhanMonica IvanC. Michael GreenliefMonica MironescuCao-An VuMonica Sorina LickerCarla F. SousaMonika Adamczyk-PopławskaCarla ZannellaMonika DąbrowskaCarles LlorMonika PrakashCarlo BieńkowskiMoonsuk BaeCarlo ContiniMor Nadia Lurie-WeinbergerCarlo GenoveseMorten LindbækCarlo PallottoMouloud KechaCarlo PerisanoMourouzis PetrosCarlo TasciniMozaniel OliveiraCarlo VallicelliMrinal BhattacharjeeCarlos Alberto Lobato TapiaMudassar Iqbal ArainCarlos BalsalobreMuhammad Adnan AshrafCarlos CaroMuhammad Ahsan NaeemCarlos Daniel Gornatti-ChurriaMuhammad ArbaCarlos David Grande TovarMuhammad Attiq RehmanCarlos Fernando MourãoMuhammad Hammad ButtCarlos Leonardo Céspedes AcuñaMuhammad Khalid KhanCarlos Martín ArdilaMuhammad Mubashar IdreesCarlotta MontagnaniMuhammad NabiCarmela MustoMuhammad NawazCarmelo BiondoMuhammad QasimCarmen ChifirucMuhammad RasoolCarmen LiMuhammad SalmanCarmen Soria-SegarraMuhammad ShafiqCarmen TeodosiuMuhammad Usman QamarCarol MaddoxMuhammad Wajid UllahCarole CreuzenetMuhammed Shafeekh MuyyarikkandyCaroline PissettiMuhammed Yunus BektayCatalina Rey-ValeirónMukesh VermaCatherine Loc-CarrilloMukti Ram PaudelCatherine MulliéMukund P. TantakCatherine Plüss-SuardMukund TantakCattaneo DarioMuralidhar TataCécile PhilippeMuralikrishna LellaCecilia PozziMuriel Primon-BarrosCelia BustosMurugesh KandasamyCéline MongaretMustafa SevindikCemil KürekciMutshiene EkwanzalaCengiz GökbulutMyeong-Gyu KimCésar LázaroMyron ChristodoulidesChad StratiloNadeem UllahChadi AbbaraNadege Lubin-GermainChaima Alaoui JamaliNadezhda FursovaChairmandurai AravindrajaNadine LemaîtreChaitany Jayprakash RaoraneNagah ArafatChander Singh DigwalNagaia CiacciChandra MadasuNagamalai VasimalaiChandran SivashankarNagaraju KerruChang-Tong YangNagaraju SakkaniChangzhen WangNagavendra KommineniChao ChenNagehan Desen KöycüChao WuNaina M. P. MaideenChaoling ChenNajma MemomCharalampos LoutradisNallely Rivero-PerezCharity Wiafe AkentenNam Su KuCharles CC LeeNamdev S. TogreChendong HanNamdev Shivaji TogreChenghung LeeNan JiangChengming WangNancy D. HansonChengxi ZhangNancy De BriyneCheng-Yen KaoNaoko YanagisawaChenjian GuNaomi OhtaChennaiah AndeNaouel KlibiChenxi LiuNarakorn KhunweeraphongCheryl Ingram-SmithNarcis I. PopescuCheuk Lun LiuNaren Gajenthra KumarChhedi Lal GuptaNarendar DudhipalaChia LeeNatalia DrabińskaChiara PagliucaNatália MartinsChia-Ru ChungNatalie FilmannChie Tamamoto-MochizukiNatalie WallisChien-Ming ChaoNatallia V. DubashynskayaChih-Cheng LaiNatarajan ArunadeviChih-Chia SuNataša HulakChih-Yu ChiNatasa OpavskiChin-Chuan HungNathalia Cristina Cirone SilvaChinmay V. TikheNathalie Grace ChuaChin-Soon PhanNaveed AhmedChis Adriana AureliaNavved AhmedChishih ChuNawaf Al-MaharikChoo Yee YuNawal SerradjiChris DurojaiyeNeda Milevska KostovaChristelle Der LoughianNeel B. ShahChrister MalmbergNeeta Upendra KumarChristian BeltzerNelly MohamedChristian HobsonNemesio Villa RuanoChristina R. BourneNermeen YosriChristina S. ThorntonNgaio RichardsChristina SakarikouNguyen NhuChristina ZalaruNguyen Trong TuanChristoph BurgerNial QuineyChristophe CurtiNicholas C. K. HengChristopher A. BeaudoinNicholas SvitekChristopher AsquithNicola NanteChristopher Hamilton-WestNicola PuglieseChristopher J. DestacheNicolas De ProstChrysanthi VoyiatzakiNicolò RossiChuan Hun DingNidhi SinglaChuda ChittasuphoNidia Cristiane YoshidaChun GaoNiels CremersChun TangNihad AdnanChun-Che HuangNikhil PaiChung Thanh ChungNikol SulloChung-Cheng WangNikola UnkovićChunlei LiNikola Zlatkov KolevCihan PapanNikolaos SoldatosCintia GonzálezNikolaos SolomakosCinzia AuritiNikolay NityagovskyClaire BeckerNikolet PavlovaClaire HeneyNil PandeyClaire PressiatNilakshi BaruaClarissa BorgesNimi VashiClaudia Cobo-AngelNina Gorišek MiksićClaudia MehedintuNing HuClaudia TodaroNir StererClaudio CaprariNiranjan ParajuliClaudiu MorgovanNirmal Kumar MohakudClayton C. CaswellNisar Ul KhaliqColm O'MorainNishad Thamban ChandrikaCôme BureauNitin DhowlagharConcepción C. GonzálezNobuhiro AriyoshiCong KongNobuhiro KanemuraConnor BeeboutNobumichi KobayashiConstantin CerbuNobuyuki ShimonoConstantinos TsioutisNoemí Buján GómezCorina CiobanasuNoor KazimCorina VasileNoor WahabCornelia MirceaNooshin SohaniCornelis SmitNoriaki IijimaCorneliu TanaseNorival Santos-FilhoCorrado TagliatiNoura DosokyCostanza VicentiniNourah Z. AlzomanCostas C. PapagiannitsisNovriyandi HanifCristian FurauNuno F. AzevedoCristian TieranuNuno V. BritoCristiane Koga-ItoNur Alim BahmidCristina Bettencourt NevesNur Izyan Wan AzeleeCristina ChircovNurdjannah Jane NiodeCristina CostaNursanti AnggrianiCristina Esmeralda Di FrancescoNurudeen Olalekan OlosoCristina ForzatoObaid AfzalCristina GavriloviciObinna ObianomCristina GhiciucOgueri NwaiwuCristina LagatollaØivind BerghCristina MesquitaOla A. TarawnehCristina Mihaela GhiciucOlga C. RojasCristina Mihaela NicolescuOlga GajtkoCristina MonteiroOlga Igglessi-MarkopoulouCristina NastasaOlga L. VoroninaCristina NicaOlha MykhailenkoCristina Nicoleta CiureaOliver KalpakCristina OlivaroOlivia DorneanuCristina Pinedo-RivillaOlivia S. K. ChanCristina PitartOlja ŠovljanskiCristina VercelliOndrej KvitekCrystal MarrugantiOns BouchamiCsaba VargaÓscar BarroD. S. RamakrishnaOsman ErkmenDafne BongiornoOsmar Antonio Jaramillo-MoralesDaizy R. BatishOuti LyytinenDajana LendakOwen LeiserDalal Hammoudi HalatOwen Martin WilliamsDale QuestOzan HaliscelikDalia Anwar HamzaOzioma NwaborDalia UrbonavičienėOzren PolasekDalila Moter BenvegnúPablo Catalá-GregoriDamian PiotrowskiPablo FresiaDamien BoscPalak PatelDamir FabijanićPalmy JesudhasanDan HuPamela L. RueggDan WangPanagiota GiakkoupiDana AkilbekovaPanagiotis Karmiris-ObratańskiDana Carmen ZahaPanagiotis SimitzisDan-Alexandru TocPang-Yun ChouDanica PollardPankaj DhakaDaniel A. AbugriPankaj PandeyDaniel ArcanjoPantu Kumar RoyDaniel DemarquePaola CremonesiDaniel Echeverria-EsnalPaola GalluzzoDaniel Enosi TuipulotuPaolo Francesco ManiconeDaniel Hernandez-PatlanPaolo MonardoDaniel J. AshworthParamanandham KrishnamoorthyDaniel J. G. ThirionParamasivam NithyanandDaniel Ortega-CuellarParaskevi MitliagkaDaniel RichterPardeep KumarDaniel SantosParide GrisentiDaniel SzulczykParthasaradhireddy TanguturiDaniel T. AndersonPasquale LincianoDaniela AraújoPatrice A. MarchandDaniela BencardinoPatrice GaurivaudDaniela BraconiPatrícia PiresDaniela CejasPatrick D. FischerDaniela Dalla GasperinaPatrick NaughtonDaniela FortiniPatrick OnyangoDaniela MarascoPatrick SchwarzDaniele De MeoPatrizia ChetoniDanielle B. GrahamPatrizia FalabellaDanijela HorvatekPatrizia MessiDanijela Horvatek TomicPatrycja Zalas-WięcekDanilo D'ApolitoPattrarat ChanchaithongDanny EpsteinPaul Brandon BookstaverDanuta DrozdowskaPaul J. DysonDarbre TamisPaul JoyceDario LeskurPaul ReynoldsDarja Barlič MaganjaPaul StoodleyDarko ModunPaulina MertowskaDarlene SantiagoPaulina PecynaDarren WongPaulo CarmoDarya NovopashinaPaulo Ricardo De SouzaDasantila Golemi-KotraPavel PadnyaDave GilmorePavel S. GribanovDave MangindaanPawan KumarDavid Chávez-FloresPawel Dabrowski-TumanskiDavid CluckPaweł KowalczykDavid De SmetPaweł KrzyżekDavid ElyPawel KwiatkowskiDavid Eugenio Hinojosa-GonzálezPaweł SolarczykDavid Gómez-RíosPaweł ŚwitDavid J. BlakePawin PadungtodDavid MusokePayam BehzadiDavid ŠilhaPedro Ernesto De ResendeDavide BavaroPedro FerreiraDavide CarcionePeechanika ChopjittDawid StefaniukPeng ChenDebabrata ChowdhuryPeng GaoDebayan DeyPercy SchröttnerDebora ColangeloPetar KostešićDeborah PedonePetca Razvan-CosminDech DokpuangPeter ImmingDeepa AcharyaPeter Konstantin KurotschkaDeepak KulkarniPeter MeyerDelia HorhatPeter MwangiDelia Mirela TitPeter OelschlaegerDelia MunteanPeter PristašDelia TitPeter Seah Keng TokDeni KostelacPeter WilsonDenis NiyaziPetra LoveckáDenisa MarginǎPetros IoannouDerry K MercerPhilip W. WertzDespoina GkentziPhilippe A. GrangeDevapriya SinhaPhilippe GabantDeyao LiPhu DuongDeyse Christina VallimPier Giorgio CojuttiDhanashree LokeshPierfrancesco GrimaDhésmon LimaPierre Louis ToutainDhwani KansalPierre Philippe NguemaDiana ÁlvarezPierre-Eric CamposDiana Camelia NuţǎPietro FerraraDiana ScorpioPilar TeixeiraDianbao ZhangPing SongDiane Ashiru-OredopePing-Jyun SungDiego Francisco FacconePinyo RattanaumpawanDikdik KurniaPiotr B. HeczkoDilep Kumar SigalapalliPiotr SzwedaDima L. WhitePiya TemviriyanukulDimitra PetropoulouPiyush BaindaraDimitri PoddighePoonam MudgilDimitrios A. AnagnostopoulosPoonam PhalakDimitrios G. GeorgakopoulosPoonam SharmaDimitrios KaragiannisPoonam VinayamohanDimitris MossialosPrabhakaran Vasantha-SrinivasanDina YarullinaPradeep KumarDinesh KumarPrajna TripathiDinesh MittalPrakash SahDingqiang ChenPramod SubediDino SgarabottoPramodkumar P GuptaDiogo Rodrigo Magalhaes MoreiraPramote ChumnanpuenDirk J. Van LeeuwenPrasanth GovindanDishon MuloiPrasanth ManoharDmitriy Nikolaevich AndreevPrasun KumarDmitry A. GryadunovPravin KaldhoneDmitry VlasovPrem Haridas MenonDmytro DmytriievPrem Pratap SinghDnyaneshwar RasalePrimrose Beryl GladstoneDoaa IbrahimPrince AgbedanuDobromira ShopovaPriya NoriDobroslawa BialonskaPriyanka LahiriDolors Rodríguez-PardoPrzemyslaw GagatDominik ŁagowskiPrzemyslaw WolakDominik RadzkiPrzemysław ZalewskiiDominika SkolmowskaQaisar MaqboolDominique LescureQazi Mohd Rizwanul HaqDonald RonningQi YanDong Wook ShinQianru YangDongdong WangQing YangDongkuk LeeQingli DongDongzhu MaQingtian LiDorin-Nicolae GheorgheQiwen DongDoris RusicQonita Kurnia AnjaniDorota Grabek-LejkoQuézia Moura Da SilvaDorota Polak-JonkiszQutaiba AbabnehDorota Tichaczek-GoskaRabab MohammedDorota WojniczRabia AyubDorothea ThompsonRabiul IslamDorothee KieferRachna VermaDragan MilicevicRadka HulánkováDragana Mitić-ĆulafićRadomir SavićDragana Vasic AnicijevicRadoslaw JaworskiDuangjai TungmunnithumRadosław StarostaDuangporn PichpolRadu BotgrosDunya L. Al-DuhaidahawRadu RacovitaDurai SellegounderRafael BaptistaDusan MisicRafael Delgado RuizDusan R. MilivojevicRafael M. CouñagoDylan WallisRafael SiqueiraEamon J. DuffyRafał OgórekEbrahim KouhsariRaffaele BaioEbru UsRaffaele CapassoEdina A. Wappler-GuzzettaRafiou AgoroEdit Y. TshuvaRaghavendhar R. KothaEdmundo Lozoya-GloriaRahila HafeezEdmundo Rosales-MayorRahman Shah Zaib SaleemEdoabasi U. McGeeRahul GauttamEdoardo CarrettoRahul TiwariEdoardo RellaRaja ChowdhuryEduardo Alvarez DuarteRaja KalluruEduardo Festozo VicenteRaja VeerapandianEduardo RodriguezRajalakshmanan EswaramoorthyEdwin Barrios-VillaRajamohanan K. PillaiEdyta B. HendigerRajesh Kumar SinghEdyta MakuchRajib BandopadhyayEfaq NomanRajneesh JaswalEffarizah Mohd EsahRakesh K. TiwariEfstratios MainasRaktham MektriratEfthimia PetinakiRaluca DumacheEfthymia ProtonotariouRaluca IsacEgidia G. MiftodeRamendra Pati PandeyEkaterina FinkinaRamesh MukkamalaEkaterina PrikhozhdenkoRamiro López-MedranoEkta MenghaniRamon RochaElastria WiditaRamunas AntanaitisElena Adelina TomaRania HathoutElena Alexandra AlexaRanjeet DhokaleElena AvdeevaRanjeev Chrysanth PulleElena B. AverinaRanjith Kumar RajendranElena De VecchiRaphaël E. DuvalElena DinteRaquel G. SoengasElena Emilia BabeşRaquel TorrijosElena GirichRareş Mircea BîrluţiuElena MateiRashmi KumariyaElena N. StrukovaRattapon UppalaElena V. EmelyanovaRaúl Carrera CerritosElena V. OrlovaRaúl Cuauhtémoc Baptista RosasElena-Suzana Biriș-DorhoiRavesh SinghEleni KaratzaRǎzvan PetcaEleni PalliRăzvan-Ionuț PopescuEleni VergadiReda Ben MridEleonora NicolaiReena Lamichhane KhadkaElham Khorasani BuxtonRehab Mahmoud Abd El-BakyEliana Carolina VesperoReham WasfiEliana Dell'olmoReinaldo GeraldoElisa BarbieriReinhard LängerElisa BindaReinhard ZelenyElisa BogossianReinis RutkisElisa MassellaRenan Dal-FabbroElisabete MachadoRenan DanielskiElisabetta BertelliniRenata SoutoElise CuquemelleRenato B. PereiraEliza MatuszewskaRenato KovacsElizabeth FernandesRene KadenElizabeth Ramírez-FloresRia BenkőElizabeth Ramirez-MedinaRicardo SilvaElizaveta PanfilovaRiccardo BartolettiElpidius J. RukambileRiccardo BientinesiElson Santiago De AlvarengaRiccardo MavigliaElvira RozhinaRichard H. ParrishElwy AshourRichard ParrishElżbieta KamyszRifat Ullah KhanElzbieta SzaconRimadani PratiwiEmanuele PontaliRimantas DaugelaviciusEmeli MånssonRita MencucciEmil PaluchRitesh SevalkarEmili DiazRitesh ThakareEmilia GaldieroRitu BanerjeeEmilia IłowskaRizwan WahabEmília SánchezRobert AncuceanuEmilio Garcia CabreraRobert BonomoEmily M. EichenbergerRobert CharvatEmine AlpRobert J. PalmerEmma Rezel-PottsRobert McLeanEmma SweeneyRobert SusloEmmanouil MagiorkinisRobert TroticEmmanuel NjogaRoberta IbbaEndre JakabRoberta Maria AntonelloEng Guan ChuaRobertas JuodkaEngy ElekhnawyRoberto Da RosEnikő BarabásRoberto VázquezEnrique Domínguez-ÁlvarezRoberto Vazquez-MunozEpaminondas ZakynthinosRobin KinnelErcan BursalRobin KöckEric AuclairRodolfo MauceriErica Valadares De Castro LevattiRodolfo ParadaErick Gutiérrez-GrijalvaRodrigo Cuiabano Paes LemeErick Martínez HerreraRodrigo FerreiraErik T. YuklRodrigo MonteiroErin M. GaffneyRohit GuptaErlia NarulitaRohit JainErshuai ZhangRok FrlanEsen SokulluRoland KlassenEsmeralda JuárezRoman LesykEsrat Jahan RupaRomána ZelkóEssam AbdelfattahRomeo T. CristinaEsther CulebrasRomeo Theodor CristinaEto Silas FernandesRomina NabieeEugene V. RadchenkoRonit SionovEugenia Silva-HerzogRosa AlduinaEulàlia Sans-SerramitjanaRosa BellavitaEurico LimaRosa GaglioneEva CunhaRosa Maria Guillen FretesEva M. Gálvez BuerbaRosalino Vázquez-LópezEva PinhoRosanna InturriEva Sánchez-HernándezRosanna RuggieroEvandro WatanabeRosario CultreraEvangelia KoukakiRosario OlivaEvangelos AxiotisRosemary Furlan-DanielEvangelos KritsotakisRosilda Mara MussuryEvann E. HiltRoy A. KhalafEvdoxia KyriazopoulouRúben FernandesEwa KarwowskaRubén L. Rodríguez-ExpósitoEwa MajewskaRuben RosalesEwa Olchowik-GrabarekRüdiger Von Eisenhart-RotheEwa SzczukaRudolf KuklaEwelina WójcikRuichao PengEzekiel GreenRuifu YangEzzat KhanRuijie HuangFabiana Dal PozzoRuipu MuFabio Del DucaRumyana MarkovskaFabio Matos ChiarelliRuojun WangFábio Muniz De OliveiraRupak NagraikFabio ZobiRupesh KumarFabiola Miranda-SanchezRuth Lindizyani MfuneFabrice CompainRuvini PathiranaFacundo RuizRyaan El-AndariFadis F. MurzakhanovRyohei NomotoFaiz Ullah KhanRyszard LauterbachFan ZhangS. Karthick Raja NamasivayamFangyuan ZhangS. M. Lutful KabirFanta Desissa GutemaSaad AlghamdiFariba DonovanSaba RiazFarideh HosseinkhaniSabina PurkrtovaFaten El SayedSabina SblanoFátima De Cássia Oliveira GomesSacha KuilFátima Galán-SánchezSadeeq Ur RahmanFauna HerawatiSadia ShakeelFausto CatenaSadri ZnaidiFazlurrahman KhanSafwan OmranFederica CarraturoSagar AryaFederica ChiapporiSajal K. SahaFederica Dall'OglioSaki GotoFederica Di CesareSalim AlbukhatyFederico PeaSalleh AnnasFederico RiuSally DemirdjianFederico SireciSalvatore BarrecaFei YanSamar ImbabyFeiyue TengSamavath MallawarachchiFelicia RuffinSambit MishraFelix BergmannSameh ElhadyFengjui ChenSami M. Abd ElwahabFengxia YangSamina AkbarFeras El-HajjiSamo FokterFernanda Maria Marins OcamposSamo JevericaFernanda Raya TonettiSampurna ChatterjeeFernanda TonelliSamuel DontaFernando Augusto Lima MarsonSamuel J. Martínez-DomínguezFernando Martínez-MoralesSamuele CerutiFernando Navarro GarciaSanath KumarFernando ReyesSanbo QinFerri Elida NoraSandeep KumarFevzi BardakciSandeep Kumar SinghFilbert TotsinganSandeep ThannaFilip ŠupljikaSándor GondaFilippo FavalliSandra Oramas-RoyoFilippo PuenteSandra PintoFilomena FortinguerraSandra PirasFiona Jane RadcliffSandra Rajme-LopezFirdoos Ahmad GogrySandra SánchezFlavia ZacconiSandra SoaresFleischer C. N. KoteySanghamitra NayakFlorence OkaforSanja Skaro BogojevicFlorentina-Daniela MunteanuSanja TomšićFlorin George HorhatSanjai SaxenaFoteini F. ParlapaniSanjay MarasiniFranca Möller Palau-RibesSanjucta DuttaFrancesca ManciantiSankarprasad BhuniyaFrancesca MensitieriSantosh KumarFrancesca PirasSantosh PeddiFrancesca ZottiSaptarshi RoyFrancesco BaccelliSara AlosaimyFrancesco BuonocoreSara Andrés-LasherasFrancesco Di BelloSara DominguesFrancesco Di GennaroSara El ZahedFrancesco FerriniSara GrilloFrancesco SegalaSara KhanFrancina Lozada NurSara PrimavillaFrancine Johansson AzeredoSara Salehi HammerstadFrancis MuchaambaSara ScuteraFrancisco EpeldeSara TedeschiFrancisco J. RoigSaran LotfollahzadehFrancisco Javier Alvarez-MartinezSarath VijayakumarFrancisco Javier Álvaro-AfonsoSari MattilaFrancisco PeixotoSatish GandhesiriFrancisco Ruiz-CastillaSatoshi MitaraiFrancisco SolanoSaul FrenkielFrancisco Wilker Mustafa Gomes MunizSaurabh MishraFrançois GuérinSaurabh RamBihariLal ShrivastavaFrançois TrotteinSavino SpadaroFrank KrumeichSawsan ZaitoneFranziska ErsoyScott M. HoltFrédéric GoormaghtighScott T. McEwenFredyc DíazSebastiaan WertenFrieder PfäfflinSebastian BöttgerFriederike HilbertSebastian Demyda PeyrásFriedrich-Karl LückeSebastian NiestępskiFunda BaǧcigilSebastian WawrockiGaber MoustafaSecil AbayGábor K. TóthSekar VijayakumarGábor KardosSelcuk ErdemGábor TernákSelma Soares De OliveiraGabriel D. PeckhamSelmin FrancescaGabriel Del RioSelvaraj BarathiGabriel IwonaSelvaraj Rajesh KumarGabriel TruebaSemaghiul BirghilaGabriela De Morais Gouvêa LimaSenthilkumar PalanisamyGabriela Eleonora Moeller ChávezSeongbin ParkGabriela KowalskaSerena SalomeGabriela Loan Loredana PopaSerge Alain Tanemossu FobofouGabriela PopaSerge AlfandariGabriele BiancoSergei Y. GrishinGabriele MeroniSergey BaykovGagandeep SagguSergey KudryashovGajane MartirosianSergii KrysenkoGalina ShepelkovaSergio MiglioreGalit Katarivas LevySergio OrtizGanesh JedheSergiu FendrihanGanesh SableSerhat AkcaalanGang ChenSerhii HolotaGareth McVickerSerhii VakalGargi PalSeri JeongGarima ArvikarSetshaba D. KhanyeGarrett EllwardSevdan YilmazGastón SilessSéverine BärGaurav BairwaSevero Vázquez PrietoGaurav KumarSevgi KolaylıGautam PareekSeyed Mohammad MazloomiGayatri Shankar ChilambiSeyed Mohammad Mirsoleimani AziziGee Jun TyeSeyede Raheleh YousefiGeetha SivasubramanianShafi MahmudGema CarrascoShah ZamanGenevieve André-FontaineShahneaz KhanGeoffrey HutinetShailesh KumarGeok Chin TanShakeel MowlaboccusGeorg ConradsShakira GhazanfarGeorge Cosmin NadǎşShamsaldeen Ibrahim SaeedGeorge EfthimiouShan GohGeorge FthenakisShang Yi LinGeorge MichailShangzhe XieGeorge W. LiechtiShaniko KaleciGeorgi BeevShanmugavel ChinnathambiGeorgia KournoutouShan-Shue WangGeorgios A. PappasShaobin WangGeorgios PapadopoulosSharat ChandraGerald MboowaSharon Tirosh-LevyGerard DumancasShashwati MathurkarGerard LevyShazia JamshedGergő TóthSheeba S. Manoharan-BasilGerhard JaritzShehzad Abid KhanGermán PeñalvaShiang-Fen HuangGhasem DiniShigeki KamitaniGhazala MuteebShigeki SetoGhulam AbbasShingo YamamotoGiacomo PapottoShin-ichi YokotaGiampaolo ColavitaShipra GroverGiancarlo CuadraShirley W. I. SiuGianfranco BocchinfusoShivankar AgrawalGianfranco La BellaShivdeep Singh HayerGianfranco SantovitoShivraj YabajiGiangiacomo BerettaShogo MoriGianmarco FerraraShohreh GhasemiGianmarco LugliShoukui HeGianmarco MangiaterraShuai MaoGianmario Edoardo PotoShuaiyin ChenGimeno MercedesShuhong SunGiorgia Della PollaShujaat AhmadGiorgia SulisShumaila HanifGiorgio GiorgiShuqi LiGiovanna IezziShusuke TodenGiovanni BocciaShyunji HayashiGiovanni Di BonaventuraSibhghatulla ShaikhGiovanni GalloSiddharth JaggavarapuGiovanni GhibaudoSiddharth MatikondaGiovanni PalleschiSiddhartha PatiGiridhar SekarSiddheshwar Kisan ChautheGitishree DasSilke MüllerGiulia BernardiniSílvia A. SousaGiulio SeveriSilvia Costa FigueiredoGiuseppe LosurdoSilvia D’AgostinoGiuseppe MancusoSilvia DeiGiuseppe MartelliSilvia LópezGiuseppe SignorielloSilvia MartuGiusy TassoneSimon ClarkeGlauco ChisciSimon CleggGloria GioiaSimón Pardiñas LópezGloria LafaurieSimona CaprarescuGloria María Molina-SalinasSimona Catalina StefanGloria Soberón-ChávezSimona IftimieGo AnanSimone CarradoriGopinath VenkatramanSimone DoreGraça PintoSimone GalloGregor KravanjaSimonetta D'ErcoleGregor ProsenSiva R. UppalapatiGregory CaputoSivalingam PeriyasamyGrigoris AmoutziasSivapong SungpraditGriselda TudóSivasamy SethupathyGrzegorz CieslarSlaven FalamićGuangde JiangSmitha SukumarGuedmiller OliveiraSobhi DanielGuenther KahlertSofia Costa-de-OliveiraGuglielmina Alessandra ChimientiSofia SaraivaGuido BloembergSohyun ChoGuillermo Martín-GutiérrezSoma Shekar DachavaramGülay BaysalSónia RamosGuoqing NiuSoojin JangGuoqing WangSookruetai BoonmasawaiGuru Raghavendra ValicherlaSophie FoleyGuy RostokerSophie MagréaultGyöngyvér SoósSorin DanGyorgy KerekesSorin RuginaHabib KhanSougata GhoshHae Suk CheongSouzan Vergkizi-NikolakakiHafiz-Muhammad-Umer FarooqiSpencer DurhamHagen FrickmannSravani AdusumalliHaishu LinSrdjan NikolovskiHaitham KalilSrinivas SistlaHakan DemirhindiStamatios GiannoukosHalis SüleymanStamatis KarakonstantisHamdy ShaabanStanislas Thiriet-RupertHamed NosratiStanislau BoguszHamid SolgiStanislav TerekhovHamza AlhamadStefania CantoreHan MeiStefania MarcheggianiHana WindersStefania ZanettiHang YangStefanie P. GlaeserHani N. NaseefStefano GrassoHanna Evelina SidjabatStefano Piero Bernardo CioffiHanna GerberStefano RavaioliHao Li (China)Stefano StracquadanioHao Li (USA)Stepan ToshchakovHao WangStephan BeiskenHao YangStephen C. HardiesHao ZhangStephen MshanaHao ZhouSteve FiesterHaragus HoriaSteven BatinovicHarald GenthSteven GregoryHarant HannaSteven KempHarbinder SinghSteven LohHari PegudaSteven Paul NisticòHari Prasad DevkotaStylianos ExarhopoulosHarichandra TagadSubhasree RoyHarini MohanramSubing QuHariprasada R. Kanna ReddySubrat BhattamisraHarrys Kishore Charles JacobSubrata MondalHasan NazikSudeshna SahaHassan Al-KaragolySudhir K. ShuklaHassan ChoudrySudipta PanjaHayden L. SmithSue NangHaytham WaliSuhaib MuflihHe LiuSuilan ZhengHe SunSujeet KumarHeather LaceySumeet K. SinghHeba AhmedSun-bean KimHeba ElgizawySundharraman SubramanianHee Eun KangSunghyun YoonHeike KasparSurajit BhattacharjyaHeinzpeter SchwermerSurajit ChatterjeeHelen AckersSuresh K. VermaHelena BarrasaSuresh NarayanasamyHelena FelgueirasSuresh PantheeHelena HybskáSuriya RamiahHelena NetoSusan SanchezHelia Bello ToledoSutherland MaciverHelio Junji ShimozakoSuzana OtaševićHema Prasad NarraSven RutkowskiHend AbdelhakimSvetoslav TodorovHenglong HuSveva Di FrancoHenrik C. BäckerSvjetlana RausHeping ZhengSwapnil Ganesh SanmukhHerman ViorelSwapnil SanmukhHerwig CerwenkaSwastik PhuleraHesam KamyabSwinburne A. J. AugustineHidefumi KasaiSyed Faisal ZaidiHideo KatoSyed Masud AhmedHidetada HirakawaSyed NabilHidetaka TorigoeSyed NaqviHinojal ZazoSyed Shams Ul HassanHirabayashi AkiSylwia AndrzejczukHiroaki KijimaSzymon TomczakHiroshi KikukawaTaha MenasriaHiroshi TomodaTahereh ShokohiHiroyuki KonnoTahir FarooqHisayuki KomakiTaiwo AremuHisham AltayebTakahisa YanoHitomi MizunoTakanobu HirosawaHoang Thi Minh HienTakashi AzumaHollis O'NealTakashi MisawaHomero Contreras-SalinasTakeshi YodaHongping WeiTakuichi SatoHooi-Leng SerTakumi UmemuraHoracio CabralTakuyaki AndoHoria OprisTalapan DanielaHoria PăunescuTaliane Leila SoaresHossam M. HassanTamara Pasqualina RussoHossein ZarrinfarTamara Pawlaczyk-KamienskaHsin-Yao WangTamara PlatteelHsiu-Jung LoTamis DarbreHsuanping ChangTania HenriquezHua QinTanja KosticHuan DongTanya ChhibberHuang QiuTao ChenHugo FragaTaoufiq BenaliHugo M. OliveiraTapas MandalHui SunTariq JamilHuilan YueTaru SinghHuili PangTarun UpadhyayHuiwen WangTasuku OkuiHumberto De FreitasTatyana PasechnikHung King TiongTauqeer Hussain MallhiHyukmin LeeTavan JanvilisriHyun Mi KangTaylor B. UpdegroveHyung Jong JinTed W. ReidHyun-moon BackTehmina KhanIan HardcastleTemidayo ElufisanIbne K. M. AliTeng ChiIbrahim El-SayedTerenzio CosioIbrahim ElsohabyTeresa C. CardosoIgnacio BrouardTeresa FascianaIktej JabbalTeresa GervasiIlaria De PasqualeTeresa Gonçalves RibeiroIliana Medina-RamírezTeresa VinuesaIlija CvijetićTerrence J. RavineIlyas AlavTerry Ann ElseImaël Henri Nestor BassoléTerry ElseImmacolata AnacarsoTeruya KomatsuImmaculata XessTeshome YehualaeshetImti ChoonaraThais GuimaraesImtiyaz Ahmad WaniThamer A. AlmangourIn Young YooTheresa ScheuInderbir SinghThi Thao Linh NguyenInmaculada Medina‐CalizThi Tuong Vy PhanInna LysnyanskyThobela NkukwanaInnocent AfekeThokur Sreepathy MuraliIoana Dorina VlaicuThomas C. WebsterIoannis KampourisThomas DandekarIoannis KoutelidakisThomas LodiseIoannis PartheniadisThomas Matthew TaylorIolanda Valentina PopaThomas McConvillleIonel MangalagiuThorsten KucziusIonela Andreea NeacsuThundon NgamprasertchaiIonela HoteaThundon Thundon NgamprasertchaiIonela Ruxandra SfeatcuTibor BotkaIonela-Larisa MiftodeTim MairIrena H. MaliszewskaTim SandleIrena IlicTimothy EricksonIrene Esteban-CuestaTing ZhangIrene SartiniTing-Shu WuIrene StefaniniTivadar KissIrfan RatherTiziana CassettiIrin SirisoontornTobias GorisIrina AndrievskayaTobías Manuel AppelIrina NegutTofayel AhmedIrina V. BoniTomas SimurdaIryna BoikoTomasz BogielIryna SamarskaTomasz GubicaIsaac AbuleyTomasz KosmalskiIsabel Cláudia Masson Poiares BaptistaTomasz KowalczykIsabel López TovarTomasz WypychIsabel Loreto Barroso De CarvalhoTomi ObeIsao NagaokaTomislav IvankovicIsha TanejaTomislav KostyanevIshaq AhmadTommaso FelicettiIsidro García-MeniñoTommaso LombardiIslam A. A. AliTon J. A. LoontjensIslam HusainToncho DinevIslam M. GhaziTony Le GallIsmael Montero FernandezTony SkapetisIsmail OdetokunTorgny SunnerhagenIsraa AlKadmyTripti SwarnkarIsrael NissanTristan T. TimbrookIssam HasniTruong Dinh HoaiItamar GonçalvesTsung-Ying YangItziar Chapartegui-GonzálezTsung-Yu HuangIulia-Cristina BagiuTsz Him ChowIuliana MotrescuTsz Tin YuIvan BruknerTuba BaygarIvan M. SavicTudor Lucian PopIvan PalaginTulip A. JhaveriIvan PavlovićTulip JhaveriIvan S. KholodilovTvrtko HudolinIvan ŠošaTzu-Chieh TangIvan V. NechepurenkoUeli Von AhIvan VokřálUgo CovaniIvana AleksićUlises Garza-RamosIvana CabarkapaUlises Hernandez-ChiñasIvanka DimovaUlrich Severin DischingerIvica MatićUma GautamIvo SirakovUrszula JankiewiczIvone MartinsUrszula ŁapińskaIwona RybakowskaUtpal NandiIzabela Kern-ZdanowiczValentina BaccoliniIzabela KruszelnicaValentina BudaIzabela MaluchValentina PucaIzabela Muszalska-KolosValentina Virginia EbaniIzabella Karska‐BastaValeria AllizondJaap Ten OeverValeria Di OnofrioJacek KozdrójValeria GarganoJacek LewandowiczValeria RussiniJacek WąsowskiValerie J. CarabettaJaeyoung KwonValério Monteiro-NetoJafir ShiraziValeriu LupuJagat RathodVanesa Antón-VázquezJagdish JoshiVanessa Branco PaulaJagoda PlaczkiewiczVânia M. MoreiraJaime Bustos-MartínezVanja SeregeljJakkarin LimwongyutVanner BoereJakkrawut MaitipVanya KurtevaJakub LasákVarish AhmadJakub RuszowskiVarsha JainJakub SuchodolskiVasfiye Kader-EsenJames Bowen StiehlVasiliki KoumakiJames C. HurleyVassilis J. DemopoulosJames DoubVassilis SkampardonisJames EdwardsVeera Venkat Shivaji Rama Rao EdupugantiJamie L. WagnerVeeranoot NissapatornJan JankowskiVeerupaxagouda PatilJan Naseer KaurVelaphi C. ThipeJan PaczesnyVera SadykovaJana KatuchovaVerica Aleksic SaboJanet HumeVéronique FontaineJanez CerarVéronique SinouJan-Falco WilbrandVéronique Yvette Ntsogo EnguénéJanja TrcekVesa Cosmin MihaiJanja TrčekVesna KosecJános DégiVictor AsensiJanthima MethaneethornVíctor García-GonzálezJari IntraVictor I. BandJarred PrudencioVíctor M. GonzálezJar-Yuan PaiVictoriya A. GeorgiyantsJasmine ChungViet LeJasminka TalapkoVijay K. SharmaJason PaxmanVijay KumarJasper JamesVijay Singh GondilJavad JavidniaVijay TripathiJavid Ahmad ParrayVijaya Anand ArumugamJavier Eduardo Garcia CastañedaVijaya Saradhi MettuJavier. CampaniniVijeta SharmaJay N. PatelVikas KumarJayanta Kumar PatraVikram SarpeJaydeep YadavViktoria RajtukovaJayedul HassanVinay KumarJayesh J. AhireVinayak M JoshiJean GuardVincent ChanJean VanderpasVincent JullienJeanie MiskoVincenza GianfrediJean-Sébastien BaumannVincenzo ZammutoJean-Yves BouetVincezo CuteriJefferson E. Contreras-RoperoVinit RajJeffrey L. WattsVioleta PopoviciJeffrey W. HallVioleta ValchevaJehan ZebViorica Railean-PlugaruJelena MilovanovicVirendra YadavJenkins AbiVirginia FajtJens StrohaekerVirginija JankauskaiteJeny ShkloverVishal KumarJeremy CharriotVishalkumar G. ShelatJerzy Jan JaroszewskiVishma Pratap SurJess VergisVito BiondiJessica K. OrtwineVitor RodriguesJessica M. CottreauVittorio MazzarelloJesús G. ZorrillaWael A. Al-ZereiniJesús Martín-GilWael ElfeilJesus Munoz RojasWael HegazyJesús TamarizWafaa A. Abd El-GhanyJevrosima StevanovicWalaa A. AlshareefJhih-Hang JiangWalaa NegmJian XuWaldemar TejchmanJiangchuan ShenWaleed Al-MarzooqiJianping MaWan Sinn YamJiaqi MiWan-Kyu LeeJie ChangWasan KatipJie GaoWasim S. El NekidyJiemin LiuWattana PelyunthaJieun KimWee Yin KohJilei ZhangWei ChengJimena MuciñoWei Ching KhorJing CongWei Guang SeetohJing GuoWei XiaoJingen ZhuWei-Chih ChenJirapas JongjitwimolWei-Hung ChengJiru HanWenchao LuJitendra TripathiWenchao YangJiyun LiWenjie WangJoakim BjerketorpWenqiang SunJoan Gómez-JunyentWen-Sen LeeJoana RoloWentao HuangJoanna FedorowiczWiesław KacaJoanna KozłowskaWieslaw SwietnickiJoanna Kwiecińska-PirógWieslawa DuszynskaJoanna Pławińska-CzarnakWilliam Gustavo SganzerlaJoanna StefanskaWilliam Hidalgo BucheliJoanna WojtackaWilliam N. SetzerJoão MesquitaWilliam SchwanJoão NevesWilliam SganzerlaJoão PaisWilliam ZaragozaJoao Ruggero NetoWilman CarrilloJoão SantosWilmer Gianfranco Silva-CasoJoe A. FralickWinfried KernJoel EllwangerWingLueng WongJohn D. ChisholmWissem MnifJohn K. JacksonWitold UhrynowskiJohn SorensenWiwat ChancharoenthanaJoice Tom JobWojciech EliaszJolanta KarakulskaWojciech GlinkowskiJolanta KutkowskaWojciech JelskiJolanta WawrzyniakWojciech SmułekJolein G. E. LaumenWolfgang BäumerJon Birger HaugWolfram MittelmeierJon P. WoodsWon Min ParkJonathan BrittonWon Y. LeeJonathan G. FryeWon-Bae ParkJonathan SellarsWoraphot TantisiriwatJong-Sup BaeWoubit AbdelaJorge Alberto García-FajardoXavier DuranJorge LopezXiao HuJorge OlivaresXiaojiong JiaJorge PadrãoXiaoju HuJosé Antonio Cervantes-ChávezXiaoliang WangJosé Augusto Ramírez TrujilloXiaolun SunJose BuesoXie BingJosé F. Gómez-ClavelXin LiJosé Luis Lázaro-MartínezXin YinJosé Luis MartínezXingao ZhangJose María Rodríguez-CallejaXinhui LiJose Oñate-GarzónXinxing ZhangJosé Rafael De AlmeidaXu YangJosé Weverton Almeida BezerraXucheng HouJose-Antonio Mirón-CaneloXuesong LiJoseph B. McPheeYafei DuanJoseph GroffYaichiro OkuzuJoseph KauerYakhya DieyeJoseph Michael Ochieng' OduorYamil LiscanoJosh SharpYaming JiuJosipa BukićYanan WangJosko MarkicYangtian YiJosy Anteveli OsajimaYannick RossezJouman HassanYan-Ping ZhuJovana FrancuzYanwen XiongJózsef SókiYaochun YuJuan A. AyalaYaoxiang LiJuan C. Vázquez-UchaYapei ZhangJuan Carlos Ángeles-HernándezYaseen AbbasiJuan Carlos Crespo-RivasYash RavalJuan Carlos Gea-BanaclocheYasuhiko MatsumotoJuan Carlos Vázquez-UchaYaswanth KuthatiJuan Jose AcevedoYazed AlsowaidaJuan LagoYazed Saleh AlsowaidaJuan Rodrigo SalazarYevgen KarpichevJuan-Pablo Gomez-EscribanoYi Long TohJugal DhandhukiaYi WangJuhee AhnYibo QiuJulia D. Toscano-GaribayYi-Chou HouJuliana FernandesYihenew Simegniew BirhanJuliana Ferreira-StrixinoYijia ZhangJuliana GarciaYijie DengJulie GorhamYiming WangJulija ArmalyteYin Ping WongJulio César Rivera-LeyvaYin ZhaiJun KuritaYin-Chou HsuJung SunwooYinghui WangJung-Chul ParkYitang SunJung-Woo BaeYlva PerssonJunhao ZhuYogesh NarkhedeJunhong WeiYogitha N. SrikhantaJun-ichi WachinoYolanda CajalJunying MaYong GuoJürgen BartelYongchel AhnJurgita ŠvedienėYonghong ZhangJussi HirvonenYong-Il KimJustin R. LenhardYongrong ZhangJustyna Karkowska-KuletaYoshiro SakaiJustyna Mazurek-PopczykYosra A. HelmyJustyna PalecznyYoung-Mog KimJustyna Staninska-PiętaYousef NaserzadehJuta KroičaYoussef AttiaJyoti MehtaYu CaiK. SamratYu KoyamaKabiru AkinyemiYuanyuan SiKacper LiberaYuanzhe LiKae HaradaYucen XieKai GuoYugal Kishore MohantaKaitlin ForsbergYuhan HaoKaja KupnikYuhki SatoKajal GuptaYuichi MurakiKalaiselvi SenthilYukihiro HamadaKalaivani ParamasivanYulia I. DeryabinaKalman ImreYulian KonechnyiKálmán ImreYulian TumbarskiKalukapuge Asanka SanjeewaYuLian WeiKamal Ahmad QureshiYuliya CherednichenkoKamaleldin SaidYu-Lung HsuKameron Y. SuginoYunqian DaiKamil JurczyszynYunsong LiuKamila KorzekwaYuriy Vasil'evKamila ZdenkovaYuting YuanKanate ThitiananpakornYu-Wei LiuKanchan BhardwajYves ReingewirtzKanchan KohliZaara SarwarKandeepan KarthigesuZahid MajeedKannan SridharanZaiga Nora-KrukleKarel HrichZakaria HafidiKarin SchwaigerZakir KhanKarol SikoraZanthia WileyKarol SteckiewiczZapryana R. DenkovaKarolina BierowiecZbigniew AdamiakKarolina ChilickaZeeshan RafiKarolina WódzZenon PogorelićKarri A. BauerZeynep GülayKartika Afrida FauziaZhaolong YuKashish MehraZhenglu WangKasi VegesanaZhengnian LiKassapa EllepolaZhenwei YuKasturi BanerjeeZhi RuanKatalin SzaboZhigang QiuKatarina D. NovovićZhijun WangKatarzyna BączekZhimin GuoKatarzyna Baldy-ChudzikZhiming ChenKatarzyna Kucwaj-BryszZhongfang TanKatarzyna KurzatkowskaZhongwei NiuKatarzyna PobiegaZhuo WangKatarzyna Wolny-KoładkaZia Ul MustafaKate A. HendricksZiad MoussaKateřina NedbalcováZihua WangKatharina RentschZikai HaoKatherine Vande PolZlatko JanječićKátia Cristina PinelloZobia NoreenKatie AldredZofia Urbańczyk-LipkowskaKato HideoZoi AthanasakopoulouKatrina BrowneZoltán BánócziKatsuya FuchinoZoran DzolicKaushalendra ChaturvediZorana KovacevicKaushik BiswasZornitsa MitkovaKavya AnnuZrinka ŠtritofKayla R. StoverZulfan ZazuliKazeem Adeyemi



